# Relationship between sublingual varices and hypertension: a systematic review and meta-analysis

**DOI:** 10.1186/s12903-024-03982-8

**Published:** 2024-02-15

**Authors:** Hosein Eslami, Fatemeh Halimi Milani, Fatemeh Salehnia, Negar Kourehpaz, Katayoun Katebi

**Affiliations:** 1 Department of Oral and Maxillofacial Medicine, Faculty of Dentistry, Tabriz University of Medical Sciences, Tabriz, Iran; 2 Faculty of Dentistry, Tabriz University of Medical Sciences, Tabriz, Iran; 3 Research Center for Evidence Based Medicine (RCEBM), Tabriz University of Medical Sciences, Tabriz, Iran

**Keywords:** Tongue disease, Hypertension, Meta-analysis

## Abstract

**Background:**

Previous research has investigated the connection between sublingual varices (SV) and cardiovascular disease, aging, and smoking. However, it is still unclear whether arterial hypertension affects the presence of SV. This meta-analysis aimed to investigate the relationship between hypertension and the presence of SV.

**Methods:**

The literature search was performed using PubMed, Web of Science, Scopus, Google Scholar, and Embase for cross-sectional studies until July 2023. PRISMA guidelines were used for article selection. A meta-analysis using standardized mean differences by a random effects model was conducted to pool studies.

**Results:**

A total of 568 articles were retrieved, of which twelve were included in the meta-analysis. Cumulatively, 2543 samples in the case group (1185 with hypertension) and 3897 samples (821 with hypertension) were studied in the control group. Using the random effects model, the pooled odds ratio (OR) revealed a significant association between hypertension and sublingual varices (OR = 2.66; 95% CI: 1.69–4.18).

**Conclusion:**

The meta-analysis showed a significant and positive association between sublingual varices and hypertension. SV’s presence could be used by dentists as a non-invasive indicator of hypertension screening.

**Supplementary Information:**

The online version contains supplementary material available at 10.1186/s12903-024-03982-8.

## Background

Sublingual varices (SV) are a local dilatation of veins often visible on the ventral surface of the tongue, though they can also be seen rarely on the floor of the mouth and lips [1, 2]. SVs are usually distributed bilaterally from the posterior region to the tip of the tongue [1, 3]. The presence of vein enlargement under the tongue characterizes SV. Sometimes, this condition is known as “caviar tongue.” Several factors, including venous structure and connective tissue disorders, have been identified as potential causes [4]. The most commonly affected intraoral areas are the sublingual area, the buccal mucosa, and the retro commissural mucosa [5]. Regarding clinical features, sublingual varicose veins are usually multifaceted, irregular, raised, or bubble-like in the ventral and lateral border of the tongue and are usually blue or purple [2, 6–8]. Lesions are mostly asymptomatic and discovered through routine clinical examinations [6]. The occurrence of SV in the general population ranges from 1.5–16.2% [4, 9]. SV is more dominant in seniors [10, 11], and age is an underlying etiological factor for SV [12–16]. SV affects both men and women [6, 17]. In addition to gender and age, other risk factors, such as smoking, hypertension, diabetes, and use of dentures, were also investigated [4, 12, 18–21]. 

Hypertension is the most common risk factor for cardiovascular disorders, affecting approximately 1.28 billion adults globally [22–24]. A large percentage of people with hypertension remain undiagnosed, untreated, or undertreated [25, 26]. Early detection of hypertension through screening increases awareness for those at risk of hypertension, and it allows for timely intervention and management of the condition [25]. 

According to international clinical practice guidelines, the risk of cardiovascular disease rises with an increase in systolic and diastolic blood pressure [27]. On the other hand, according to the WHO, 46% of adults with hypertension are not informed of their condition [28, 29]. Blood pressure is measured using systolic blood pressure (SBP) and diastolic blood pressure (DBP). The current guidelines for categorizing high blood pressure are as follows: stage 1 hypertension is defined as an SBP ranging from 130 to 139 or a DBP ranging from 80 to 89, and stage 2 hypertension refers to an SBP ≥ 140 or a DBP ≥ 90 [30–32].

Some studies have found a significant association between SV and hypertension [17, 21, 33]. A study with an eight-year follow-up reported that participants with SV showed a higher prevalence of hypertension than participants with no SV. Meanwhile, some studies did not reveal an association between SV and hypertension [16]. Considering the different and controversial results of published studies, developing an accumulative result of studies could provide a valuable guide, especially for the timely and effective diagnosis of hypertension. Therefore, this study aimed to systematically review the relationship between sublingual varicose veins and high blood pressure.

## Methods

### Protocol and registration

This is a systematic review and meta-analysis study conducted in 2023. PRISMA guidelines were used to report the results. This study is registered in PROSPERO (CRD42023476936).

### Eligibility criteria

Articles with cross-sectional or case‒control designs in English published until July 2023 were included. Quasi-experimental, letters to the editor, commentary, and conference abstracts were excluded.

### Information sources and search strategy

The PEO approach was applied to establish the clinical inquiry in the following way: P (population): all patients; E (exposure): hypertension; O (outcome): sublingual varices. The PubMed, Web of Science, Scopus, Google Scholar, and Embase databases were searched, as well as related journals and article references. The following MeSH and free terms are used in different combinations: sublingual varices, lingual varices, hypertension, and high blood pressure. The exact search strategy is available in Supplementary 1.

### Study selection and data extraction

The Endnote X8 software package was used to organize and screen retrieved citations. First, duplicates were excluded, and then articles were screened by reviewing their titles to exclude nonrelevant citations. Screening through abstracts was the second step, which excluded some articles not reporting the needed information. The remaining articles were investigated based on inclusion and exclusion criteria through their full text. Two researchers (KK and FHM) ran the screening process independently and consulted a third reviewer (HE) for disagreements. An extraction table was used to extract data from the included studies. The author’s name, publication year, country, sample size in case/control groups, case/control groups age and sex, and number of samples with and without hypertension, both in case/control groups, were the data items that were extracted.

### Risk of bias assessment

The Joanna Briggs Institute (JBI) Checklist for Cross-Sectional Studies was used to assess the studies’ quality and risk of bias. The reporting bias in this systematic review was independently assessed by two authors (FHM and KK) and the discrepancies were discussed with a third author (HE).

### Statistical analysis

The pooled measure of association, odds ratios (ORs), with a 95% confidence interval (CI), was calculated for the relationship between varices and BP using the random effects model. For pooling systolic and diastolic blood pressure, the weighted mean differences (WMD), endpoint scores, or change scores were used to represent the difference in BP between groups. The BP values in mmHg were compared between study groups, including patients with varices and controls. Cochran’s Q test and I^2^ were used to assess heterogeneity between studies. Sensitivity analyses were conducted for results with heterogeneity. The subgroup meta-analysis by continent was used for the association between varices and BP. STATA 14.0 (Stata Corp, College Station, TX, USA) was used for meta-analysis.

##  Results

**Fig. 1:**
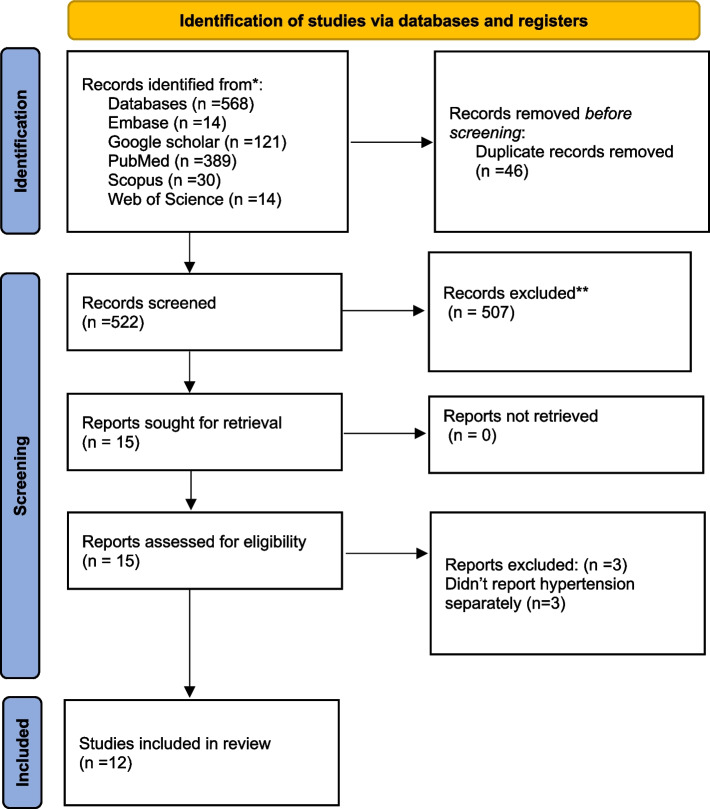
The screening process of retrieved articles

A literature search resulted in 568 articles, of which 46 were duplicates. After carefully screening the retrieved articles, 12 were eventually included in the meta-analysis. The PRISMA flowchart for article screening is reported in Fig. 1.

Cumulatively, 2543 samples were in the case group, and 3897 were in the control group. Regarding the share of samples with hypertension, 46.5% (*n* = 1185) of samples in the case group and 21% (*n* = 821) had hypertension. The characteristics of the included studies are detailed in Table 1.

**Table 1:** Characteristics of the included studies

N	Author, date	Country	Sample size case/control	Mean Age	Sex (%F) case/control	Hypertension frequency in case/control	Normotensive frequency in case/control
1	Accardo et al. (2021) [1]	Italy	724/284	45–85	59.4/57	518/227	206/57
2	Akkaya et al. (2019) [21]	Turkey	186/505	NR	67.2/683	63/23	123/482
3	Bergh et al. (2022) [33]	Sweden	326/663	Case: 68.8 ± 7.3 Control:65.3 ± 7.2	48.5/57.5	136/179	190/484
4	Hedström et al. (2015) [48]	Sweden	114/317	55.3 ± 10.9	56.4	57/63	57/254
5	Baharvand et al. (2022) [37]	Iran	91/60	47.58 ± 12.19	49	33/4	58/56
6	Jafari et al. (2022) [6]	Iran	271/207	74.5	50.2/67.6	103/74	168/133
7	Shivakumar et al. (2020) [17]	India	65/136	52.3 ± 11.5	52.3/33.8	45/30	20/106
8	González-Álvarez et al. (2022) [20]	Spain	162/336	NR	70.4/69.9	50/52	112/284
9	İçöz et al. (2021) [49]	Turkey	155	38.8	60.6	22/35	133/431
10	Olufemi et al. (2016) [50]	Nigeria	31/169	51.6 ± 0.9	44.5/44.5	28/72	3/97
11	Jamali et al. (2023) [38]	Iran	109/391	Case: 43.3 ± 11.3 Control:42.7 ± 12.2	45/56	84/58	25/333
12	Lazos et al. (2020) [2]	Argentina	309/363	18–92 (median = 37.7)	61.8/58.6	46/4	263/359

*NR* Not Reported

### Risk of Bias assessment

According to the JBI Checklist, all 12 articles had a low risk of bias. Table 2 displays the risk of bias in the studies included.

**Table 2:** Risk of Bias of the included studies

	Q1	Q2	Q3	Q4	Q5	Q6	Q7	Q8	Q9	Final score
Jafari et al.	Y	Y	Y	Y	Y	Y	N	Y	Y	7/8
Accardo et al.	Y	Y	Y	Y	Y	Y	Y	Y	Y	8/8
Akkaya et al.	Y	Y	Y	Y	Y	Y	Y	Y	Y	8/8
González-Álvarez et al.	Y	Y	Y	Y	Y	Y	Y	Y	Y	8/8
Bergh et al.	N	Y	Y	Y	Y	Y	Y	Y	Y	7/8
Hedström et al.	Y	Y	Y	Y	Y	Y	Y	Y	Y	8/8
İçöz et al.	Y	Y	Y	Y	Y	Y	Y	Y	Y	8/8
Jamali et al.	Y	Y	Y	Y	Y	Y	Y	Y	Y	8/8
Lazos et al.	Y	Y	Y	Y	Y	Y	Y	Y	Y	8/8
Baharvand et al.	Y	Y	Y	Y	Y	Y	Y	Y	Y	8/8
Olufemi et al.	Y	Y	Y	Y	Y	Y	N	Y	Y	7/8
Shivakumar et al.	Y	Y	Y	Y	Y	Y	Y	Y	Y	8/8

Y: Yes, N: NO, Q1: Were the criteria for inclusion in the sample clearly defined? Q2: Were the criteria for inclusion in the sample clearly defined? Q3: Were the study subjects and the setting described in detail? Q4: Was the exposure measured in a valid and reliable way? Q5: Were objective, standard criteria used for measurement of the condition?Q6: Were confounding factors identified? Q7: Were strategies to deal with confounding factors stated? Q8: Were the outcomes measured in a valid and reliable way? Q9: Was appropriate statistical analysis used?

### Meta-analysis

**Fig. 2:**
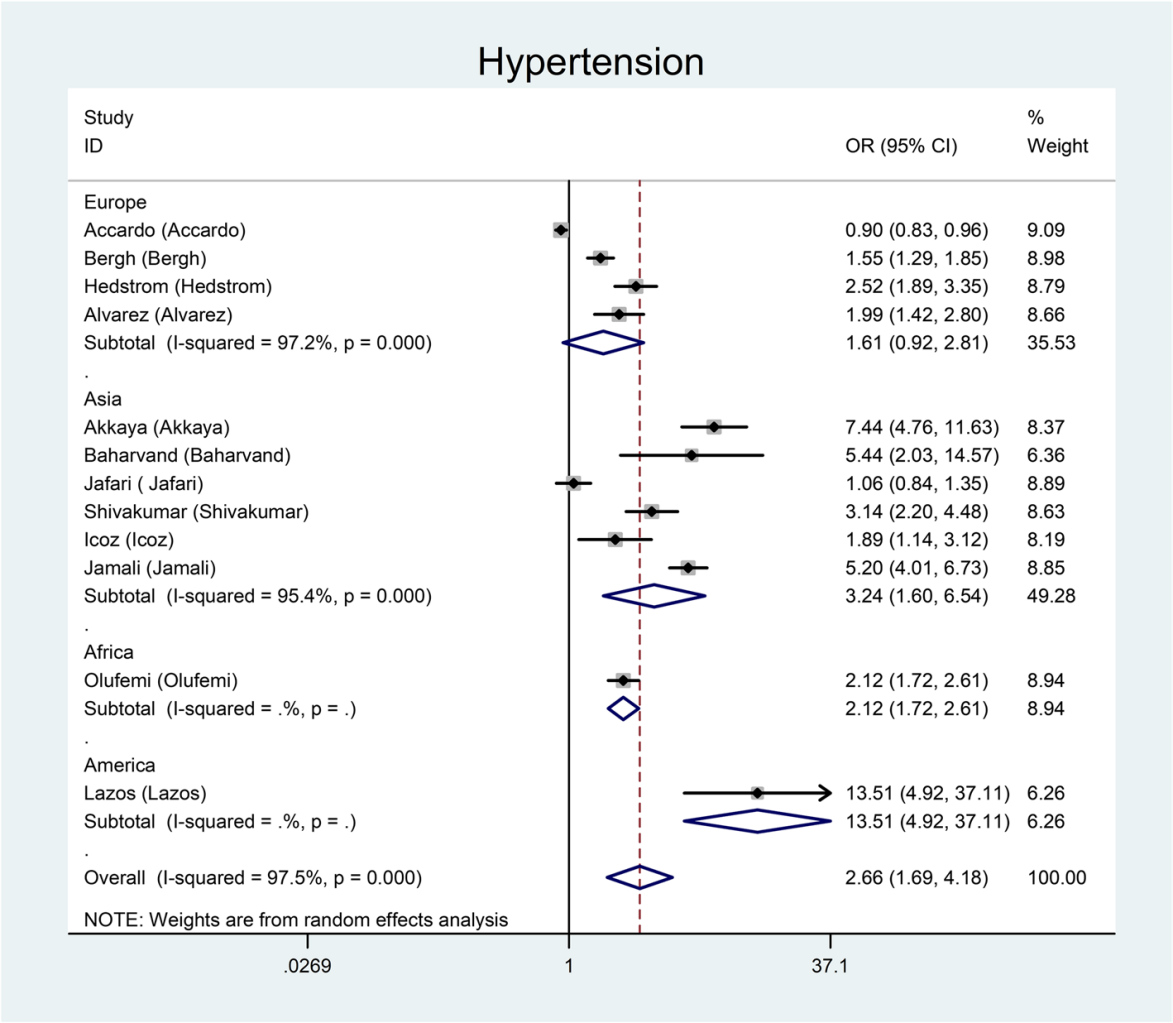
Meta-analysis of the association between BP and sublingual varices

Figure 2 represents the overall and subgroup meta-analysis results for the relationship between BP and the risk of sublingual varices using the random effects model for ten studies. Pooled OR using a random effects model showed a significant association between BP and sublingual varices. High BP increased the risk of varices 2.66 times compared to healthy controls (pooled OR = 2.66; 95% CI: 1.69–4.18).

In the subgroup analysis by continent, the minimum and maximum measures of associations were found for Europe (pooled OR = 1.61; 95% CI: 0.92–2.81) and America (Argentina) (pooled OR = 13.51; 95% CI: 1.92–37.11), respectively.

### Sensitivity analysis

**Fig. 3:**
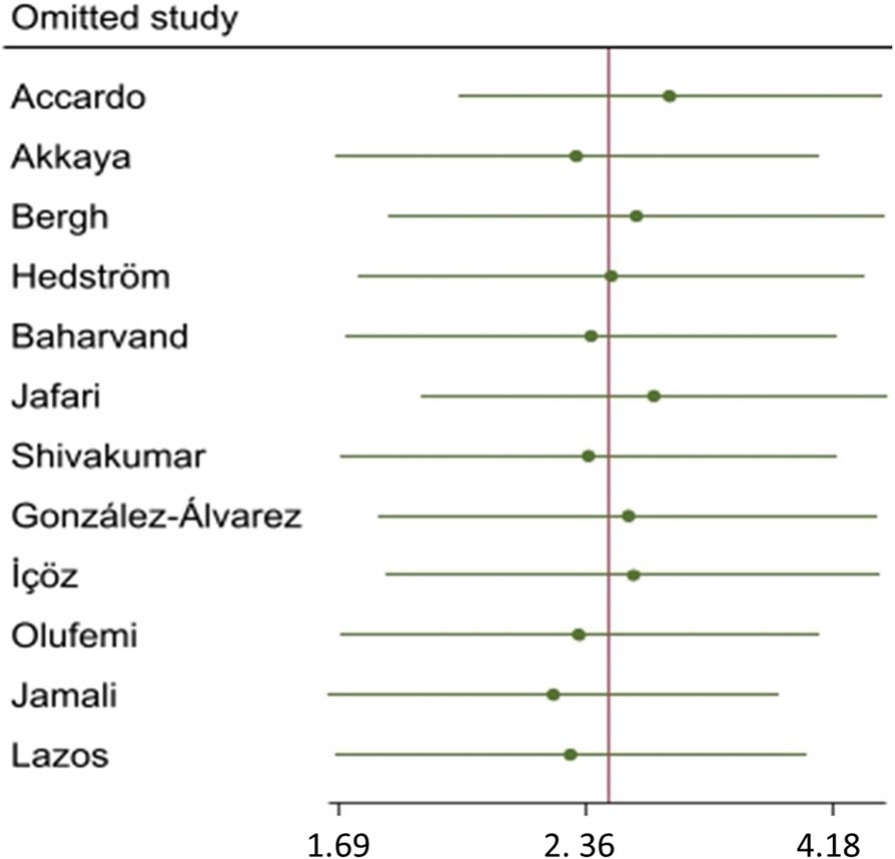
Sensitivity analysis to examine the effect omitting individual studies on odds ratio (OR)

Sensitivity analysis was performed by excluding studies one-by one (Fig. 3). It was demonstrated that the omission of each study did not influence the pooled odds ratio significantly, suggesting that the result was relatively robust.

**Fig. 4:**
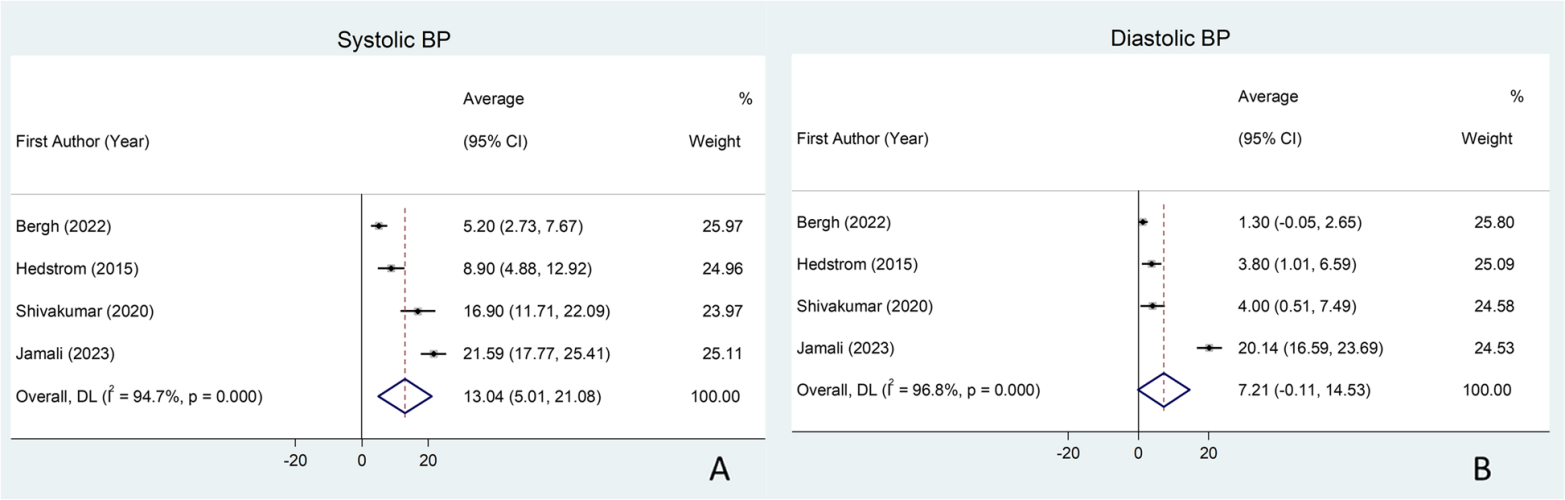
Meta-analysis for the association between systolic (**A**) and diastolic (**B**) blood pressure and sublingual varices

Figure 4 shows the meta-analysis of the weighted mean difference of SBP and DBP in sublingual varices and controls using a random effect model. The WMD of SBP and DBP were increased in sublingual varices (WMD = 13.04; 95% CI: 5.01–21.08) and (WMD = 7.21; 95% CI: − 0.11–14.53), respectively (Table 3).

**Table 3:** Characteristics of studies reporting SBP and DBP for subgroup meta-analysis

N	First Author	SBP in case	SBP in control	DBP in case	DBP in control
1	Bergh et al. (2022) [33]	139.5 ± 18.6	134.3 ± 18.8	85.4 ± 9.9	84.1 ± 10.8
2	Hedström et al. (2015) [48]	132.1 ± 19.3	123.2 ± 17.3	83.4 ± 13.3	79.6 ± 12.2
3	Shivakumar et al. (2020) [17]	138.1 ± 18.6	121.2 ± 15.1	85.2 ± 12.5	81.2 ± 10.2
4	Jamali et al. (2023) [38]	139.68 ± 19.01	118.09 ± 13.78	100.45 ± 17.81	80.31 ± 12.08
5	Lazos et al. (2020) [2]	140.96	127.98	86.9	79.38

*SBP* Systolic blood pressure, *DBP *Diastolic blood pressure

##  Discussion

Cardiovascular diseases (CVDs) have the highest burden of disease and are the leading cause of death worldwide [39]. According to the WHO, hypertension is the major risk factor for CVDs and the cause of premature death [40]. The literature indicates that hypertension could result in organ damage. Therefore, timely diagnosis and treatment of hypertension is vital for the prevention of its complications [41, 42]. 

Meta-analysis results showed that the weighted mean difference of both systolic and diastolic blood pressure was higher in adults with sublingual varices (*p* = 0.000). Hedstrom et al., in their clinical study, reported a significant difference in systolic and diastolic blood pressure between patients with grade 0 and grade 1 SV. Similarly, a study by Bergh et al. with the participation of 989 patients indicated higher mean systolic blood pressure (139.5 mmHg vs. 134.3 mmHg) for patients with SV. The circulatory anastomosis in the tongue’s venous system or a hemodynamic impact in which the artery pressure affects the veins through arteriovenous shunts may be the cause of the link between clinical alterations in sublingual varices and hypertension [1, 4, 37]. 

Some included studies have reported a significant relationship between hypertension and sublingual varices [10, 38, 43]. A Lynge et al. study on elderly individuals showed a significant relationship between hypertension and SV frequency. Hedstrom et al. reported a significant association between hypertension and SV, as they reported that SV grade 1 prevalence increased from 21.8% among participants with no hypertension to 30.8% in patients with hypertension stage 1 and 43.6% among patients with stage 2 hypertension. Accardo et al. discovered a correlation between SV and compensated and resistant hypertension, but no correlation was found in newly diagnosed cases. This could be because resistant and compensated hypertension had been present for a more extended period, leading to vascular damage that was not seen in newly diagnosed cases. No positive correlation between SV and hypertension was shown for this study in the current meta-analysis. This could be because of combining all three groups of studies (compensated, resistant, and newly diagnosed) into one and considering them as a single group with hypertension.

This meta-analysis revealed a significant relationship between hypertension and the presence of SV, which could be used as a screening method for hypertension in dental settings. This could highlight dentists’ role in hypertension screening in collaboration with the public health system. Subgroup analysis showed that odd ratio was higher in Asia compared to the Europe. This could be due to life style or genetic differences [45]. Dentists are among the first healthcare providers likely to encounter early signs and symptoms of systemic disease and play an essential role in diagnosing and effectively managing the situation. The pathophysiological background of this association remains unclear. Nevertheless, it is believed that high blood pressure in arterial hypertension may play a role in the onset or exacerbation of sublingual varices. The raised pressure in the arterial system could cause venous congestion and expansion of the sublingual veins. Some experts suggest that this may be because of circulatory anastomosis in the venous system of the tongue [3]. Another theory is that it may be due to a hemodynamic effect, where the arterial pressure affects the veins through arteriovenous shunts [46]. Increased arteriovenous blood flow could transfer arterial pressures which is much higher than venous pressure to the venous circulation, with vein dilatation and consequent morphologic changes in their walls. The negative staining to glucose transporter protein − 1 is consistent with the hypothesis that SV result from structural alterations [47]. However, further research is needed to clarify the mechanisms underlying this correlation.

A positive predictive value of 0.5 and 0.80 of negative predictive value was reported for sublingual varices as a detection sign for hypertension [33]. The results of this meta-analysis showed that SV is a common benign clinical sign of hypertension and could be used as a valuable measure to screen people regarding hypertension. Therefore, the role of dentists in screening undiagnosed hypertension or undertreated cases is valuable. Lazos et al. reported an association between SV and hypertension using only two grades (none or few visible varices vs. medium or severe varices) to classify sublingual varices, and this may lead to bias due to patient classification overlap with other studies [3]. 

Aging, one of the confounding factors for the results of this study, wasn’t reported separately for each group in some of the included articles. Therefore, an analysis wasn’t possible. The study by Baharvand et al. didn’t find any relationship between age and SV [37]. However, Akkaya et al. reported that sublingual varices are associated with aging [21]. Shivakumar et al. found a positive correlation between SV and hypertension in patients older than 40 years old [17]. It seems that the effect of age should be considered in the interoperation of results about the relationship between sublingual varices and hypertension.

### Limitations

The included studies had a case-control or cross-sectional design, which has limitations. Many of the included studies didn’t report the mean age of the participants separately for each group, and also some studies didn’t report the number of females and males in each group. Therefore, conducting a subgroup analysis for these two important factors wasn’t possible and this might be the reason for heterogenicity between the studies. Only English articles were included in this systematic review which rises a language bias. One of the limitations of this study is related to the population of included studies related to their genetics and nutritional status, which wasn’t reported, and smoking history which wasn’t matched in some studies. All these factors could affect hypertension status.

##  Conclusions

The meta-analysis showed a significant and positive association between sublingual varices and hypertension. The risk of sublingual varices was 2.66 times higher in patients with hypertension. Moreover, both systolic and diastolic blood pressures were higher in patients with SV. Although results should be interpreted with caution, this could be used as evidence for a probable diagnosis of undiagnosed hypertensive cases. Dentists, as health team members, could use this recommendation to take part in hypertension screening and advise their patients with SV to be screened for hypertension measures. Prospective cohort studies provide more substantial evidence, which could lead to the development of clinical guidelines for better and more effective management of hypertension.

### Supplementary Information

**Supplementary Material 1.**

## Data Availability

No datasets were generated or analysed during the current study.

## Abbreviations

SV: Sublingual varices
SBP: Systolic blood pressure
DBP: Diastolic blood pressure
ORs: Odds ratios
CI: Confidence interval
WMD: Weighted mean differences
CVDs: Cardiovascular diseases
