# Correction: Relationship between sublingual varices and hypertension: a systematic review and meta-analysis

**DOI:** 10.1186/s12903-024-04372-w

**Published:** 2024-05-21

**Authors:** Hosein Eslami, Fatemeh Halimi Milani, Fatemeh Salehnia, Negar Kourehpaz, Katayoun Katebi

**Affiliations:** 1 Department of Oral and Maxillofacial Medicine, Faculty of Dentistry, Tabriz University of Medical Sciences, Tabriz, Iran; 2 Faculty of Dentistry, Tabriz University of Medical Sciences, Tabriz, Iran; 3 Research Center for Evidence Based Medicine (RCEBM), Tabriz University of Medical Sciences, Tabriz, Iran

**Correction to: BMC Oral Health (2024) 24:240**

10.1186/s12903-024-03982-8.

In the sentence beginning “Lazos et al. reported” in this article, the text “Lazos et al. reported an association between SV and hypertension using only two grades (none or few visible varices vs. medium or severe varices) to classify sublingual varices, and this may lead to bias due to patient classification overlap with other studies” should have read as “Lazos et al. reported an association between SV and hypertension using a different classification, unlike others who used only two grades (none or few visible varices vs. medium or severe varices) to classify sublingual varices, and this may lead to bias due to patient classification overlap with other studies”.

