# Supplementary material for: Relationship between sublingual varices and hypertension: a systematic review and meta-analysis
Source: BMC Oral Health. 2024 Feb 15;24:240. doi: 10.1186/s12903-024-03982-8 (PMC10868067; doi:10.1186/s12903-024-03982-8)
Supplement: Supplementary file 1 — Supplementary Material 1. [file 12903_2024_3982_MOESM1_ESM.docx]

('sublingual varices':ti,ab OR (sublingual:ti,ab AND ('varicosis'/exp OR 'microvaricosity':ti,ab OR 'phlebarteriectasia':ti,ab OR 'phlebectasia':ti,ab OR 'phlebectasis':ti,ab OR 'portal varix':ti,ab OR 'prevaricose syndrome':ti,ab OR 'prevaricosis':ti,ab OR 'pyeloureter varix':ti,ab OR 'ulcus varicosum':ti,ab OR 'varices':ti,ab OR 'varicose complex':ti,ab OR 'varicose syndrome':ti,ab OR 'varicose ulcer':ti,ab OR 'varicose vein':ti,ab OR 'varicose vein syndrome':ti,ab OR 'varicose veins':ti,ab OR 'varicosis':ti,ab OR 'varicosity':ti,ab OR 'varix':ti,ab OR 'varix ulcer':ti,ab OR 'vein ectasia':ti,ab OR 'vein varicosis':ti,ab OR 'venectasia':ti,ab OR 'venous varicosis':ti,ab OR 'venous varix':ti,ab))) AND ('hypertension'/exp OR 'htn (hypertension)':ti,ab OR 'acute hypertension':ti,ab OR 'arterial hypertension':ti,ab OR 'blood pressure, high':ti,ab OR 'cardiovascular hypertension':ti,ab OR 'controlled hypertension':ti,ab OR 'endocrine hypertension':ti,ab OR 'high blood pressure':ti,ab OR 'high renin hypertension':ti,ab OR 'hypertension':ti,ab OR 'hypertensive disease':ti,ab OR 'hypertensive effect':ti,ab OR 'hypertensive response':ti,ab OR 'neurogenic hypertension':ti,ab OR 'preexistent hypertension':ti,ab OR 'salt high blood pressure':ti,ab OR 'salt hypertension':ti,ab OR 'secondary hypertension':ti,ab OR 'systemic hypertension':ti,ab)

( ( ( TITLE-ABS-KEY ( sublingual )  AND  TITLE-ABS-KEY ( "varicosis"  OR  "microvaricosity"  OR  "phlebarteriectasia"  OR  "phlebectasia"  OR  "phlebectasis"  OR  "portal varix"  OR  "prevaricose syndrome"  OR  "prevaricosis"  OR  "pyeloureter varix"  OR  "ulcus varicosum"  OR  "varices"  OR  "varicose complex"  OR  "varicose syndrome"  OR  "varicose ulcer"  OR  "varicose vein"  OR  "varicose vein syndrome"  OR  "varicose veins"  OR  "varicosis"  OR  "varicosity"  OR  "varix"  OR  "varix ulcer"  OR  "vein ectasia"  OR  "vein varicosis"  OR  "venectasia"  OR  "venous varicosis"  OR  "venous varix" ) ) )  OR  ( TITLE-ABS-KEY ( ( "sublingual varices" ) ) ) )  AND  ( TITLE-ABS-KEY ( "hypertension"  OR  "htn hypertension"  OR  "acute hypertension"  OR  "arterial hypertension"  OR  "blood pressure, high"  OR  "cardiovascular hypertension"  OR  "controlled hypertension"  OR  "endocrine hypertension"  OR  "high blood pressure"  OR  "high renin hypertension"  OR  "hypertension"  OR  "hypertensive disease"  OR  "hypertensive effect"  OR  "hypertensive response"  OR  "neurogenic hypertension"  OR  "preexistent hypertension"  OR  "salt high blood pressure"  OR  "salt hypertension"  OR  "secondary hypertension"  OR  "systemic hypertension" ) )
